# International biathlon season during the COVID-19 pandemic was based on frequent on-site PCR-testing protocol with rapid result management

**DOI:** 10.3389/fspor.2023.1217463

**Published:** 2023-08-31

**Authors:** Katja Mjøsund, Mahinour Ghaleb, Lars Kolsrud, Jim Carrabre, Florian Kainzinger, Daniel Boehm, Felix Bitterling, Bernd Wolfarth

**Affiliations:** ^1^ The International Biathlon Union (IBU), Salzburg, Austria; ^2^ Paavo Nurmi Centre and Unit for Health and Physical Activity, University of Turku, Turku, Finland; ^3^ Think.Health Hygiene Solutions, Berlin, Germany; ^4^ Norges Skiskytterforbund Oslo, Norway; ^5^ Department of Sports Medicine, Humboldt University and Charité University School of Medicine, Berlin, Germany

**Keywords:** COVID-19, biathlon, SARS-CoV-2, pandemic, sports event, prevention, respiratory infection

## Abstract

The resumption of professional sports during the COVID-19 pandemic has been described in team sports but less in individual sports. The International Biathlon Union implemented a COVID-19 concept for the 2020–2021 season aimed to mitigate the risks of transmission by rules designated for the professional biathlon environment. The “bubble” model was based on regular reverse transcript polymerase chain reaction (PCR) testing with rapid results and efficient result management protocol. The objective of this study was report incidence and transmission of SARS-CoV-2 among professional biathletes and staff undergoing frequent PCR testing and risk reduction measures during the international season 2020–2021. The efficiency of risk mitigation measures was also evaluated based on the incidence data. During the 4-month season, altogether 22,182 SARS-CoV-2 PCR tests were conducted on all individuals participating in international biathlon season (athletes, team staff and organizing committee). Ninety-six (0.4%) PCR tests were positive and 30% of the positive PCR tests were considered “persistent positive” following recovery from a recent COVID-19 infection. No transmission events were detected following contact with “persistent positive” cases during the season. A great majority of the positive PCR tests were recorded during the first days after arrival in the “bubble”, often in the first entry test taken by the on-site laboratory. In conclusion, a “bubble model” based on frequent PCR testing and hygiene rules was efficient in keeping the infection rate low. The competition activity including international travel was safe, and most of the infections seemed to originate from outside of the “bubble”.

## Introduction

Coronavirus disease 2019 (COVID-19) is a highly infectious disease caused by severe acute respiratory syndrome coronavirus 2 (SARS-CoV-2) (1). The global COVID-19 pandemic led to a significant social and economic disruption all over the world, including postponement of large sports events. Professional sport provides cultural, economic and employment benefits. During the pandemic, sports organisations assessed the health risk associated with an event based on event size, sport risk, community transmission/prevalence and geographical location, and implemented risk reduction and mitigation measures in keeping with WHO and best practice guidance. Typically, this was done in a stepwise fashion, with timeframes and restrictions varying markedly across nations and sports systems (2). The resumption of competition and training activity has been described in professional level team sports such as soccer, football, hockey, basketball and baseball but to a smaller extent in professional level individual sports (2–9). In professional golf, several risk reduction and mitigation measures on players and staff have been succesfully implemented enabling return to competition (10).

Biathlon is an winter endurance sport combining cross country skiing and shooting. The International Biathlon Union (IBU) organizes two main series: the World Cup (WC) as a series 37 competitions and the second-tier IBU Cup. During the 4-month season the event circuit travels from country to country with only some breaks. In addition to the 40 national teams (300 athletes and 350 staff members) and IBU staff (100), there are approximately 400 media representatives as well as 500 volunteers at every event. In the pre-pandemic era up to 30.000 spectators visited the events daily.

Due to the long continuous season with a large travelling group of international participants, the biathlon WC season is different from many of the previously reported “bubble models” with the aim to secure a single game or a shorter series of games between a limited number of teams, or an event at a single location. The purpose of this study was to report incidence and transmission of SARS-CoV-2 among professional biathletes and staff undergoing frequent regular PCR testing and risk reduction measures during the international season 2020–2021. The efficiency of risk mitigation measures is also evaluated based on the incidence data.

## 2. Methods

### 2.1. Study design

This is a retrospective observational study of the IBU COVID-19 concept during the 4-month long international competition season 2020–2021 during the COVID-19 pandemic. The main focus is on the IBU Biathlon World Cup but also results from the lower-tier series IBU Cup and Youth and Junior World Championships are reported. The efficacy of the COVID-19 concept is assessed by the results of PCR-tests of all participants in the events. Furthermore, the incidence of SARS-CoV-2 positive PCR tests in elite biathletes competing internationally 4 months during the COVID-19 pandemic is reported.

### 2.2. Subjects

Anonymized PCR test data from all participants of the IBU season were included; altogether 22.182 samples from 3,400 subjects (645 athletes, 485 staff members, 710 media representatives and 1,560 organization committee volunteers or IBU officials were analysed.

### 2.3. The transmission risk reduction measures (“IBU COVID 19 concept”)

For the 2020–2021 season the number of events was reduced by staying two weeks in one location instead of weekly competitions at different venues, to reduce travel. When possible, the travel between venues in different host countries was organized by designated IBU charter flights.

Those travelling by car (mostly ski service van drivers) were instructed to avoid contacts during travel.

**Figure 1:**
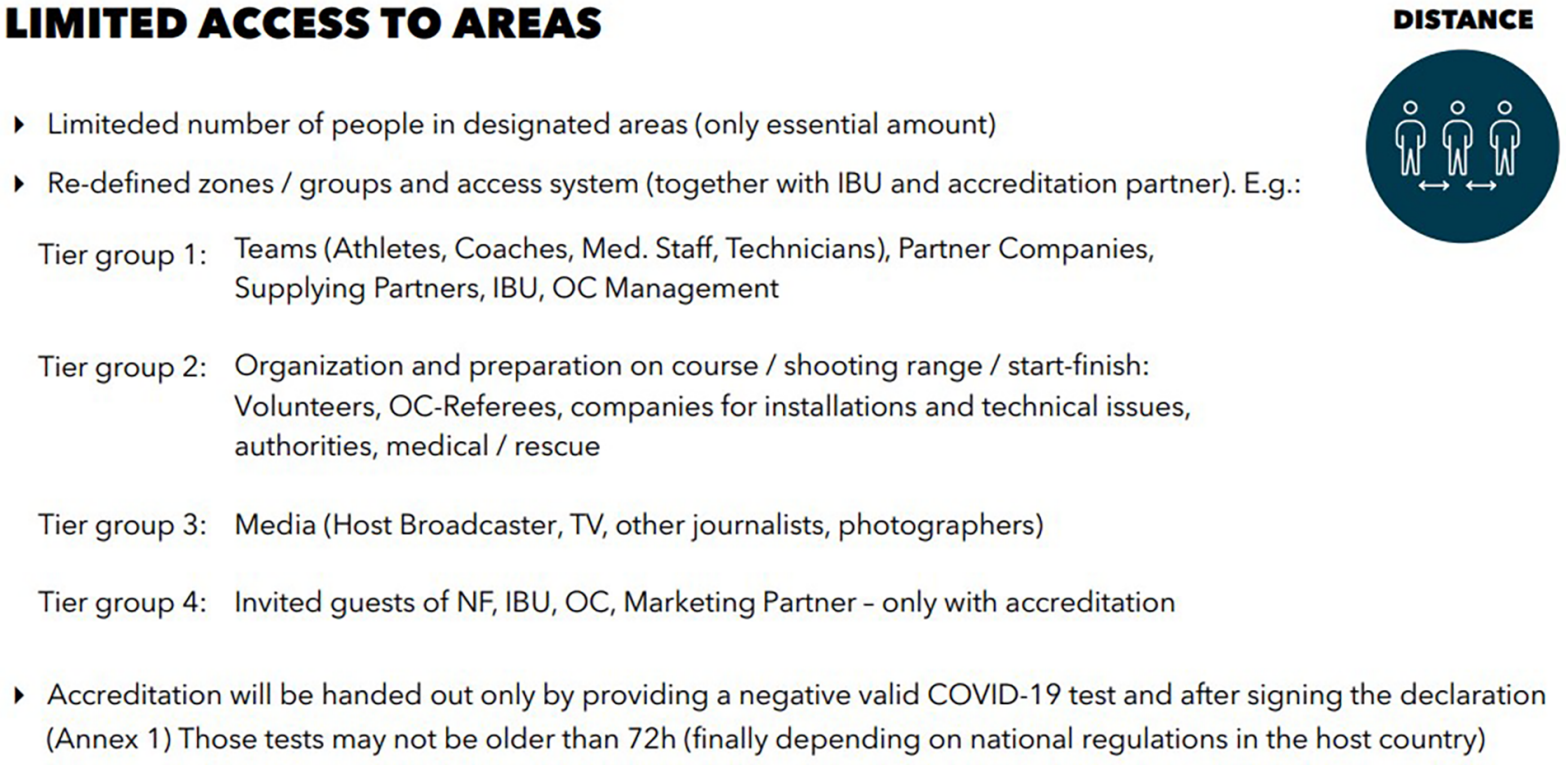
Tier group system. OC, organization committee; NF, national biathlon federation.

**Figure 2:**
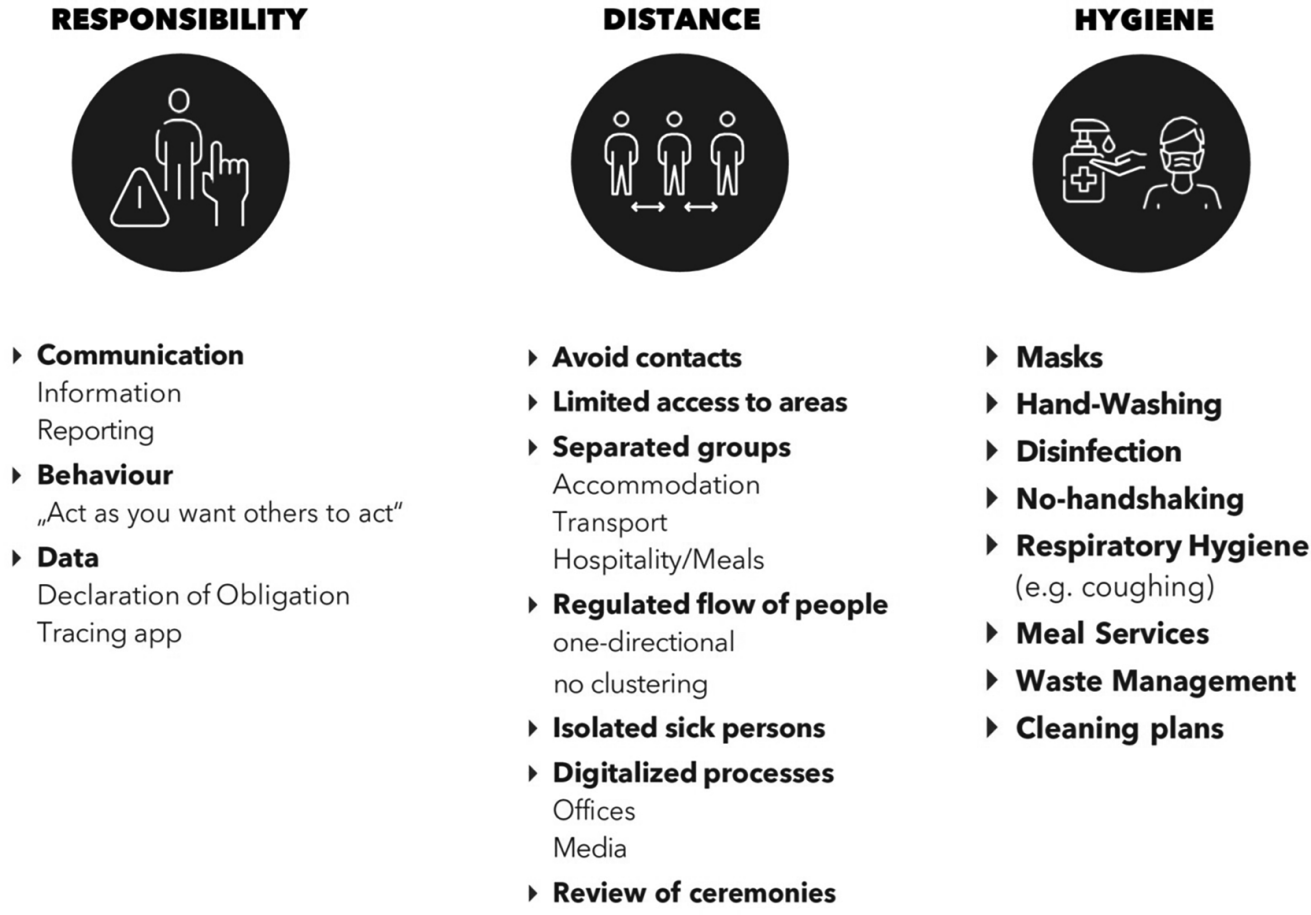
Overview of the IBU hygiene concept.

The number of participants in the venues was limited to absolute minimum with the exception to athletes, whose participation was similar to an ordinary season. The subjects were divided into tier groups (Figure 1), which were kept separated from each other during all events. No spectators were allowed at IBU events and all the events were broadcasted on TV. All subjects had to comply with risk reduction measures called “IBU COVID-19 Concept” (Figure 2) and signed a declaration accepting the rules and consequences of violation, respectively.

A test protocol was implemented by the IBU in collaboration with a mobile laboratory (Think.Health Co., Berlin, Germany) and the local OCs, to provide regular PCR testing of the participants every 3–4 days throughout the season, except for the first World Cup event in Kontiolahti, Finland due to national regulations.

A designated IBU COVID-19 team was responsible for immediate handling of test results and had a direct contact to the teams as well as the local health authorities for reporting results, handling of quarantine as well for assistance for immediate medical care is needed.

The IBU concept (Figure 2) instructed mandatory use of surgical or FFP2 mask except while eating and during exercise. In some countries, FFP2 mask was required by the authorities. The team Tier Group was accommodated in hotels with no other guests. The hotels were encouraged to maintain a high level of hygiene, and the rooms were cleaned only when the Teams were not present.

### 2.4. PCR testing

For each sample two nasal swaps were taken: the first one for a pooled analysis and the second for the verification of the possible positive test.

The PCR pooling method was used where up to 10 samples were pooled to a fast track real-time (RT) PCR analysis. The analysis was performed using Spindiag fully automated Rhonda Test System, (Spindiag, Freiburg, Germany) using a two-step PCR procedure, “nested PCR” using a broad gene marker set within the E-gene of the SARS-CoV-2 virus. The Cycle threshold (Ct) values up to 18 were considered positive cases potentially in the main phase of the infection and thus with possible transmission of infection. The average PCR run time was 50 min, which secured rapid turnaround times on site (1–2 h).

Due to local legislative regulations, during World Cup Finland, the local licensed laboratory (Eastern Finland Laboratory Centre Joint Authority Enterprise; ISLAB) was employed. The analysis followed their standard protocols for SARS-CoV-2 PCR testing (Allplex 2019-nCoV Assay; Seegene Inc, Seoul, South Korea).

### 2.5. Protocol for a positive PCR test result

The subject, team leader and the local authorities were immediately informed about a positive test result by the IBU COVID-19 team. The subject was immediately isolated and further team/group members were assessed by the IBU COVID-19 team to identify and isolate the contact persons immediately. The action by the IBU COVID-19 team ensured isolation, already before the authority decision, mitigating possible further transmission. The final decision on isolation was made by the local authorities according to the national rules and regulations.

### 2.6. “Persistent positives”; previously SARS-CoV-2 positive individuals that continue be tested positive at PCR

Individuals with a recent SARS-CoV-2 infection may remain (RT-PCR) positive at high cycle threshold (Ct) counts after recovery from the infection (11, 12). Subjects with a positive PCR for SARS-CoV-2 within the past three months were encouraged to send their previous SARS-CoV-2 laboratory test results to IBU ahead of their arrival, or to keep the documents ready for a rapid review and only RT-PCR results were accepted. The documents were reviewed by the IBU COVID-19 team physicians, together with the local authorities. If the individual had a positive PCR test with high Ct values, and previous PCR test(s) confirmed a SARS-CoV-2 infection within the preceding 3 months, the individual was considered “persistently positive” and no quarantine measures or contact tracing were applied.

Individuals who were PCR-positive in the IBU testing system or who were considered “persistently positive” were removed from testing protocol for a period of three months. This was done to avoid a possible new positive PCR test result in every event, which would have led to an isolation until the authorities had handled the case.

## 3. Results

The number of SARS-CoV-2 positive cases was low during the biathlon winter season 2020–2021. Altogether 22.182 SARS-CoV-2 PCR tests were performed (Table 1). Most of them were conducted at the IBU World Cup or at the IBU World Championships (altogether 15.092 PCR tests; 0.3% positive and 0.2% “persistent positive”; Table 1). At Junior World Championships, European Championships and the lower-tier series IBU cup, 7.090 tests were conducted of which 0.1% were positive with acute infection and 0.05% were assessed as “persistent positive” (Table 2).

**Table 1:** Overview of SARS-CoV-2 testing at biathlon world cup and world championship (WCH) 2020–2021.

Timeline	Biathlon world Cup/WCH locations	No. of tests	No. of persons with negative results	No. of persons with SARS-CoV-2 positive PCR (new pos. cases; persistent pos.)	Rate of positive cases	Rate of new pos. cases	Rate of persistent pos. cases
23.11.20–06.12.20	Kontiolahti - Finland	2.412	897	40 (17; 23)	4.27%	1.81%	2.45%
07.12.20–19.12.20	Hochfilzen - Austria	2.151	888	1 (1; 0)	0.11%	0.11%	0.00%
04.01.21–17.01.21	Oberhof - Germany	2.839	1.251	25 (24; 1)	1.96%	1.88%	0.08%
18.01.21–24.01.21	Antholz - Italy	1.098	597	1 (0; 1)	0.17%	0.00%	0.17%
07.02.21–21.02.21	Pokljuka - Slovenia (WCH)	3.166	1.030	13 (13; 0)	1.25%	1.25%	0.00%
28.03.21–13.03.21	Nove Mesto - Czech Rep.	2.258	761	1 (1; 0)	0.13%	0.13%	0.00%
16.03.21–21.03.21	Östersund - Sweden	1.168	716	1 (1; 0)	0.14%	0.14%	0.00%
Grand Total		15.092	6.140	82 (57; 25)	1.3%	0.9%	0.4%

**Table 2:** Overview of SARS-CoV-2 testing at IBU Cup, IBU open European championships (OECH) and youth & junior world championships (YJWCH) 2020–2021.

Timeline	IBU Cup/OECH/YJWCH locations	No. of tests	No. of persons with negative results	No. of persons with SARS-CoV-2 positive PCR (new pos. cases; persistent pos.)	Rate of positive cases	Rate of new pos. cases	Rate of persistent pos. cases
11.01.21–24.01.21	IBU Cup - Arber	1.881	566	12 (8; 4)	2.08%	1.38%	0.69%
24.01.21–31.01.21	OECH - Duszniki Zdroj	1.051	610	1 (1; 0)	0.16%	0.16%	0.00%
08.02.21–20.02.21	IBU Cup - Osrblie	896	356	0	0%	0.00%	0.00%
24.02.21–06.03.21	YJWCH - Obertilliach	2.460	1.497	0	0%	0.07%	0.00%
09.03.21–14.03.21	IBU Cup - Obertilliach	802	426	1 (1; 0)	0.23%	0.00%	0.00%
Grand Total		7.090	3.455	14 (10; 4)	0.4%	0.29%	0.12%

Altogether 96 PCR tests were positive (Table 1). Among them twenty-nine (30%) were considered “persistent positive”.

**Figure 3:**
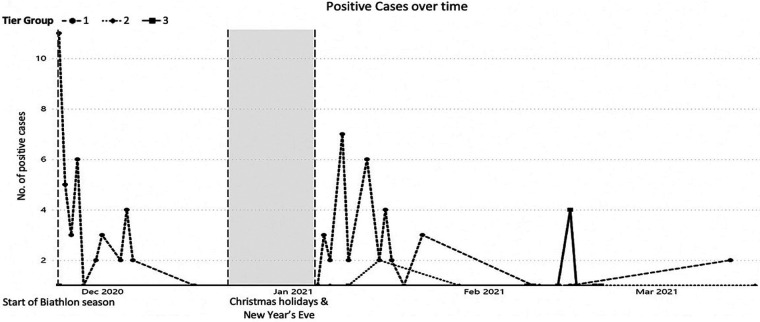
SARS-CoV-2 positive cases over time at all international biathlon union (IBU) international events during the season 2020–2021 presented by the tier groups. The tier 4 had no positive cases.

A majority of the SARS-CoV-2 positive results occurred in the entry test of the very first World Cup round in Kontiolahti (40 out of season's 96 positive results; 41.6%; Table 1 and Figure 3).

During the third and fourth week of the season in Austria), only two persons were tested positive with SARS-CoV-2. One was a person who was entering an IBU event for the first time (“entry test”) and the other was a “persistent positive” due to a recent COVID-19 infection.

Similarly, to the season start (round 1), there was a peak in the number of positive cases after a mid-winter holiday break at the entry tests to the IBU “bubble” in Oberhof (round 2). Twenty-four new PCR positive cases was 26% of the total number of positive tests during the season (Figure 3).

During the World Championships a great majority of SARS-CoV-2 positive results (10 out of 13) came from media representatives, and only one positive case was a team member at an entry test.

In the final round 3 of the World Cup season in Czech Republic and Sweden, only a total of two positive PCR tests were recorded out of 3.426 tests conducted (Table 1 and Figure 3). This was despite the difficult pandemic situation due to the high incidence rates at that time at the venue host countries.

In the first World Cup event in Kontiolahti, a large number (23) of “persistent positive” individuals with both high Ct values and documentation of recent positive PCR test, were detected.

## 4. Discussion

The incidence and transmission of SARS-CoV-2 were low among professional biathletes and support staff undergoing frequent PCR testing and risk reduction measures, throughout the international biathlon season during the COVID-19 pandemic, despite international travel and an intense competition schedule. Working in close partnership with the national authorities and the organising committees, the IBU was able to deliver all 126 competitions it had planned for the season.

The cornerstones of the model were regular PCR testing with very rapid and efficient result management followed by isolation and quarantine in collaboration with the national authorities. Also, the closed campus approach (“bubble”) and strict hygiene rules were important. In retrospect, it can be speculated if some rules such as mandatory mask outdoor, were even too cautious, as transmission risk in outdoor sports may be quite low (11, 13). However, this was not known at the time.

The efficacy of the concept was determined by the very low number of PCR-verified SARS-CoV-2 new positive cases. The majority of the positive results originated from the entry tests at the beginning of the season and after the mid-winter break, when the teams arrived from outside of the “bubble” (Table 1 and Figure 3). Especially during the mid-winter break many team members had been to social gatherings with family and friends. Along with the time spent in the “bubble”, the number of positive PCR tests decreased to almost zero, especially towards the end of the season (Figure 3) despite the difficult COVID-19 situation in the host countries Czech Republic and Sweden.

This is the first study to describe a COVID-19 prevention strategy in an individual winter sport, over a whole international season. During the season 0.016% of all individuals who were subject to testing and 0.001% of all PCR tests were SARS-CoV-2 positive which is in line or even less than the incidence in other sports implementing frequent PCR test scheme and hygiene rules. Professional golf, another individual outdoor sport, reported 2,900 RT-PCR tests performed on 195 professional golfers during the PGA European Tour across 11 countries in 2020, and only four players tested positive (0.14% of tests) showing the efficiency of risk mitigation and testing programs in individual sports (10). Although both are professional individual sports, the differences between the risk mitigation challenges between biathlon and golf include the large number of technicians in biathlon as well as the winter season requiring staying indoors.

As a professional team sport in 2020, major league basketball reported 91 of 169.143 samples (0.05%) positive; 57 of the 91 persons with positive test results were players and 34 staff members, which is very much in line with our results (14). Schumacher at al. described a truncated football season with a tailored infection control programme based on preventive measures and regular PCR testing every 3–5 days, combined with serology testing for immunity. They reported that among 1.337 football players, staff and officials over the period of 9 weeks, 85 subjects had a positive PCR test (5). The incidence was similar to the general population during the same time period in an area with a relatively high incidence of COVID-19 while in our study, the incidence within the IBU “bubble” was much lower than in the surrounding society with the clear exception of the entry to the first World Cup in Finland (4)^.^The COVID-19 prevention strategy in Deutsche Bundesliga was based on strict rules, and a 7-day team quarantine before the continuation of the season. A repeated PCR test twice a week was applied to 1.702 individuals; all players and staff members with contact to players. During the whole season, the incidence rate was low and only ten players and four officials were tested positive. Four of all positive cases were proven “persistent positives” (4). Finally, the soccer season opening during the pandemic in Denmark was based on weekly PCR test for 11 consecutive weeks. The incidence for players was 0.53% (4/748) which is higher than that in our study or in that Bundesliga (4, 6). This may indicate that once-week testing is not sufficient.

In line with other sports (4, 5, 14), a large number of the positive cases positive during the IBU season were team staff members. It is possible, that the education about infection prevention does not reach all staff members, and the incentive to follow the infection prevention guidelines may be lower in staff members than in athletes. This may be especially true for the periods at home in between the races. The staff often have family and children, which may increase the risk of transmission at home. It is of major importance that, in addition to athletes, the preventive measures and testing are directed to the whole team who work close to the athletes (coaches, physiotherapists, physicians and technicians).

In the IBU COVID-19 concept, everybody was required to present a negative pre-entry SARS-CoV-2 PCR test taken a maximum of 72 h prior to arrival into the “bubble”.

However, a negative pre-entry test alone would not have been enough to ensure safety in the “bubble” Most of the positive PCR tests were recorded by the on-site laboratory during the first days after arrival in the “bubble”, often in the first test taken on-site at the event (Figure 3). This has important implications for the planning of future events. A single negative entry test is not enough to ensure safety. In theory, a requirement of two negative PCR tests with self-isolation before the arrival could have been a solution, such as in the Olympic Summer Games in Tokyo 2021 (15). However, the frequent PCR test on-site with a very short turnaround time was another solution to ensure the safety of the participants. The infections were detected at an early stage, which minimized the risk of transmission. This finding is also supported by the low Ct-values upon the first positive PCR test for each individual, and the fact that the individuals were asymptomatic at the time of their positive test.

A vital part of the model was the on-line results management leading to immediate isolation of the SARS-CoV-2 positive individuals and their close contacts. It has been shown that test-trace-quarantine approach can control the epidemic both in theory and in practice (16), but it's success is contingent on high testing and tracing rates, high quarantine compliance, relatively short testing and tracing delays, and moderate to high mask use (16). The rapid information to individuals and teams affected mitigated the transmission risk already before the authorities were available to assess the case.

The explanation for many positive cases in the first on-site test to the event, despite negative pre-entry test at home, is not clear. Some individuals may have contracted the virus just before their arrival at the World Cup. It can also be theorized that some individuals had false negative PCR tests due to sampling errors or laboratory issues, as the quality of the laboratories from around the world could not be proven. The Olympic Games in Tokyo and Beijing only accepted PCR tests from pre-accepted licensed laboratories (15).

The participants in the IBU competitions were encouraged to report any symptoms associated with COVID-19, to self-isolate and have an extra PCR test upon symptoms. No symptoms were reported and no extra PCR tests for symptomatic individuals were asked for by the participants. This can be due to the unusually low number of respiratory infections other than COVID-19 during the pandemic or unwillingness to report respiratory symptoms. All individuals who tested positive for acute infection of SARS-CoV-2 were asymptomatic upon the routine PCR test that revealed the infection. Some individuals reported symptoms such as fever later, when already isolated (personal communication). To our knowledge, none of the individuals who tested positive at the IBU events showed symptoms of a serious systemic COVID-19 illness or required medical assistance through the event organizers. However, this was not assessed directly, and is not the main scope of this report.

Individuals with a recent SARS-CoV-2 infection may remain (RT-PCR) positive at high cycle threshold (Ct) counts after recovery from the infection (11, 12). These positive PCR results may represent low levels of replicating virus or non-infectious viral RNA fragments; however, individuals who remain persistently RT-PCR positive with high Ct values have not been observed to be infectious (6). The event participants who had been tested positive for SARS-CoV-2 within the past three months and successfully released from isolation, were considered “persistent positive” and no quarantine or contact tracing were applied. Twenty-three of the 29 tests (79%) that were considered “persistent positive” were taken during the entry to the very first World Cup event in Finland. Some of the teams had reported outbreaks of COVID-19 during training camps earlier in the season (personal communication), which may explain the high number of positive findings and clustering within some teams. These “persistent positive” cases at the entry to the first World Cup event formed clusters by country, being eight individuals from one nation and six from another and these nations reported an outbreak of COVID-19 during training camps earlier in the training season (personal communication).

No transmission was detected following contact with “persistent positive” cases during the season, based on the data. This is in agreement with the earlier findings in NBA basketball (7).

A handling strategy for persistent positive individuals is imperative when applying a test protocol in a sports setting, to avoid unnecessary isolation or exclusion from competition.

Adherence to the hygiene rules and a good compliance to the COVID-19 concept were prerequisites for the success of the model. Education of the team leaders was started by online meetings already before the season and repeated at the team captains at every event. There was a regular testing schedule with a dedicated time slot for every individual. The test results were followed-up on-line and a failed test would have led to exclusion from the event.

Many of the teams had high incentives for prevention of COVID-19 and also their own, strict COVID protocol. However, there was some reluctancy to follow the hygiene measures in the beginning of the season, mostly due to lack of education. The frequent testing and worry of being excluded from competition may have been a driver for compliance to basic hygiene protocols. It has previously been shown that frequent testing may increase the awareness of the pandemic and the adherence to hygiene rules (17).

Especially during the first part of the season, there were some rule breaches that were sanctioned. A task force group was nominated by the IBU to review possible breaches. The sanctions ranged from reprimand to a fine or exclusion from the IBU events for a certain period of time. Also, the consequences for breaches of the rules may have underlined the importance of these, and increased awareness.

In the face of the global COVID-19 pandemic, many sports events promoted the safety of the athletes and staff through different sets of tests and rules, with various outcomes (3–10). This study shows that it is possible to prevent spreading of SARS-CoV-2, a virus that can be transmitted both via respiratory droplets and aerosols, in a series of large international sports events during the most intensive period of a pandemic. The “bubble” model with frequent PCR testing and very rapid results management can be successfully implemented to mitigate the risk of transmission of respiratory viruses in sports events with large amounts of international participants.

## Data Availability

The original contributions presented in the study are included in the article, further inquiries can be directed to the corresponding author.
